# Design of push–pull system to control diesel particular matter inside a dead-end entry

**DOI:** 10.1007/s40789-015-0076-z

**Published:** 2015-08-12

**Authors:** Yi Zheng, Magesh Thiruvengadam, Hai Lan, Jerry C. Tien

**Affiliations:** Department of Mining & Nuclear Engineering, Missouri University of Science and Technology, Rolla, MO 65401 USA; Clean Air Power Inc., Poway, CA 92064 USA; Division of Mining and Resources Engineering, Department of Civil Engineering, Monash University, Clayton Campus, Wellington Road, Clayton, VIC 3800 Australia

**Keywords:** Diesel particulate matter, Computational fluid dynamics, Ventilation, Underground condition, Push–pull system

## Abstract

Diesel particulate matter (DPM) is considered to be carcinogenic after prolonged exposure. With more diesel-powered equipment used in underground mines, miners’ exposure to DPM has become an increasing concern. This paper used computational fluid dynamics method to study the DPM dispersion in a dead-end entry with loading operation. The effects of different push–pull ventilation systems on DPM distribution were evaluated to improve the working conditions for underground miners. The four push–pull systems considered include: long push and short pull tubing; short push and long pull tubing, long push and curved pull tubing, and short push and curved pull tubing. A species transport model with buoyancy effect was used to examine the DPM dispersion pattern with unsteady state analysis. During the 200 s of loading operation, high DPM levels were identified in the face and dead-end entry regions. This study can be used for mining engineer as guidance to design and setup local ventilation, select DPM control strategies and for DPM annual training for underground miners.

## Introduction

For underground mines, self-propelled diesel equipment that does not require power cable or constant charging batteries is preferred because working faces usually cover extensive areas where these facilities are not available. However, emission from the tailpipe and its subsequent distribution in the underground mine are of growing concerns for miners.

Diesel particulate matter (DPM) is the particulate by-product of diesel exhaust and it can exist in different modes with different size distributions (5 nm to 10 µm). Due to its very small size and very complex adsorb ability (more than 1800 different organic compounds and potentially toxic hydrocarbons were identified (CFR 2001)), it can be breathed into the alveolar region of the lungs of miners and cause acute health problems such as asthma, eye and nose irritation, headaches and nausea (Kahn and Orris 1988; Wade and Newman 1993; Rundell et al. 1996) to long term carcinogenic effects (NIOSH 1988; EPA 2002).

For underground coal mines, diesel engines used underground are divided into three categories under Mine Safety and Health Administration (MSHA) regulations: “permissible”, “nonpermissible heavy-duty equipment, generators, and compressors” and “nonpermissible light-duty equipment”. Equipment under each category is required to emit no more than a certain amount of DPM per hour (30 CFR 72.D 2014); otherwise, it will not be allowed to operate underground.

For underground metal/non-metal mines, MSHA regulations limit a miner’s personal exposure to DPM no more than 160 µg/m^3^ of total carbon (TC) for an average eight-hour equivalent full shift (effective from May 20, 2008) (30 CFR 57.5060 2014). Till today, there are still mines that cannot meet this regulation limit.

To control DPM hazards, two types of strategies have been commonly used. One is DPM reduction and removal before it is released from the engine tailpipe, which includes proper diesel engine selection and maintenance (McGinn 1999; Anyon 2008; McGinn et al. 2010), use of alternative fuels (Zannis et al. 2008; Bugarski et al. 2010), and exhaust gas treatment devices (Shah et al. 2007; Bugarski et al. 2009), e.g., diesel particulate filters (DPF). The other is through control measures after DPM is discharged into the environment—mine ventilation system, an enclosed equipment cab with filtered breathing air (environmental cab), personal protective equipment, and administrative controls (Cecala et al. 2005; Noll et al. 2008; MSHA 2013).

Experience (Bugarski et al. 2011) showed that no single strategy can solve all DPM problems and a combination of several measures needs to be implemented in the field to attain compliance. To achieve an effective, efficient, and economical control scheme, an understanding of DPM behaviour in mining environment can be very useful in selecting the control strategies and training the miners. Numerical simulations using CFD can be used for that purpose by visualizing DPM distribution based on laboratory experiments and field studies.

CFD simulations have been successfully used in mining research to detect spontaneous combustion and apply inertisation in gob areas (Yuan and Smith 2007; Ren et al. 2005), study airflow patterns and gas concentrations in continuous miner operations or heading development (Sullivan and Heerden 1993; Hargreaves and Lowndes 2007; Wala et al. 2007; Kollipara et al. 2012; Torno et al. 2013), investigate scrubber intake designs for longwall dust control (Ren and Balusu 2008), and estimate a mine’s damage status by tracer gas and simulation after a disaster (Xu et al. 2013). Simulation of DPM dispersion in underground mines was carried out by Zheng and Tien (2008), in which DPM was considered to behave like a gas. Subsequent study showed that it gave good quantitative agreement with practical accuracy for the DPM distribution and successfully identified the DPM affected areas above the threshold limit (Zheng et al. 2011). In the present study, DPM emission was also treated as a gas to examine its diffusion inside an underground single dead end entry.

Push–pull systems use both blower fan/tubing and exhaust fan/tubing systems, which can combine the effects of both single systems to lower the DPM levels in the dead-end face area. However, if not properly designed, the combination cannot provide the maximum outcomes and may interfere with each other. In this study, the effect of four different push–pull ventilation systems on DPM distribution inside a single dead-end entry was studied for a loading operation. CFD method was used to perform the evaluation and an optimum result was reached based on the four scenarios designed and assumptions made. This study can be used for mining engineer as guidance to design and setup of local ventilation. The high DPM regions revealed by the simulation can also be used for selection of DPM control strategies and DPM annual training for underground miners.

## Problem statement and CFD modelling

### Statement of the problem

**Fig. 1:**
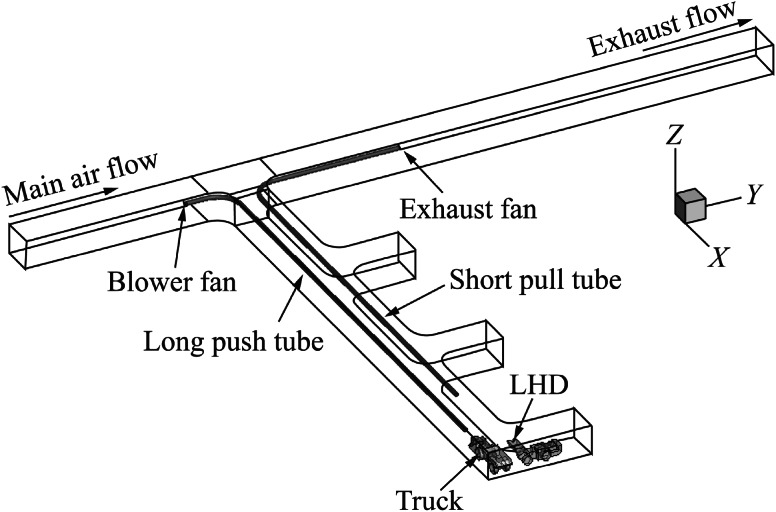
Computational domain with long push and short pull ventilation system

**Fig. 2:**
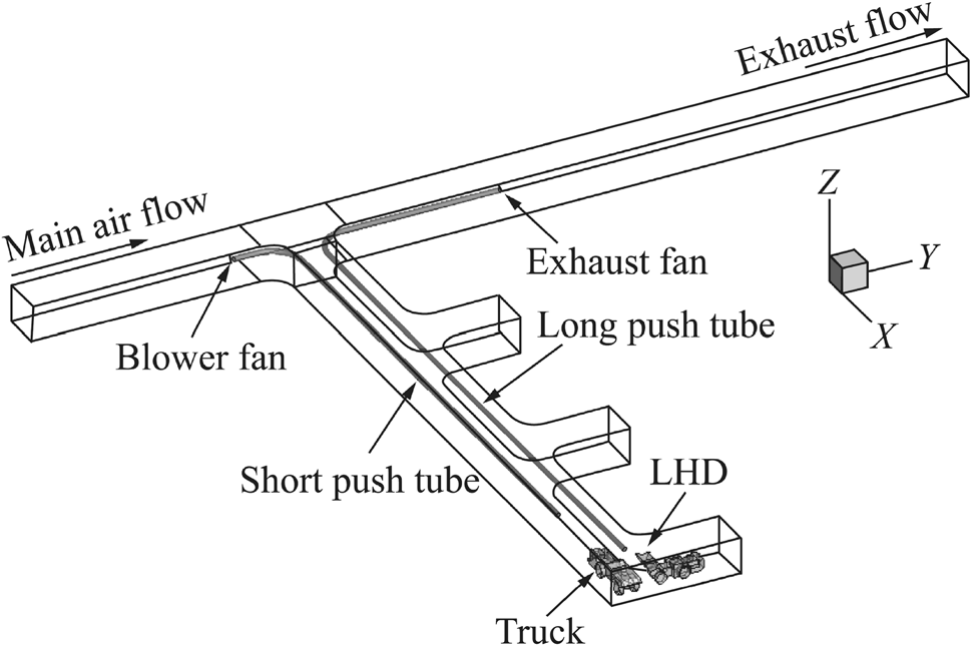
Computational domain with short push and long pull ventilation system

**Fig. 3:**
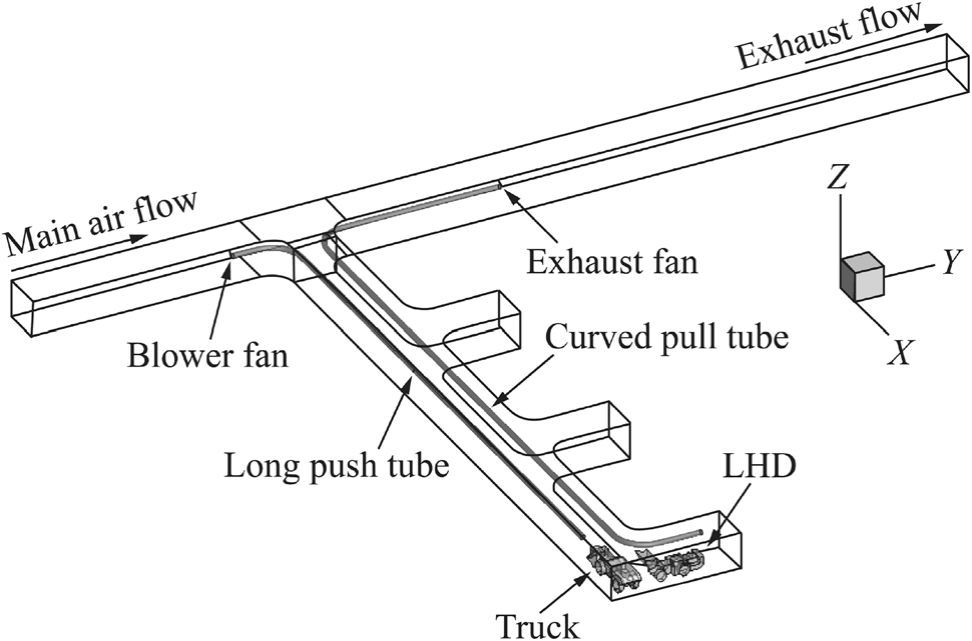
Computational domain with long push and curved pull ventilation system

**Fig. 4:**
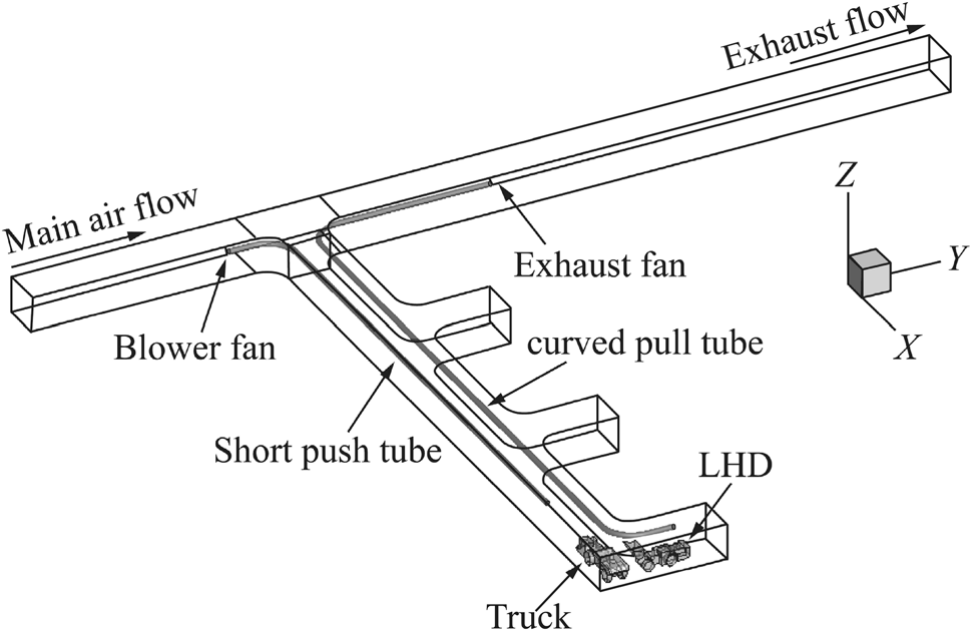
Computational domain with short push and curved pull ventilation system

A schematic of the computational domain for each push–pull ventilation design is shown in Figs. 1, 2, 3, 4. The four design cases considered in this study were: case (1), long push and short pull tubing (Fig. 1); case (2), short push and long pull tubing (Fig. 2); case (3), long push and curved pull tubing (Fig. 3); and case (4), short push and curved pull tubing (Fig. 4). The main entry measured 6 m in width, 5 m in height and 131 m in length, while the dead-end measured 6 m in width, 5 m in height, and 90 m in length. The three stub entries were evenly developed inside the dead-end entry and had the same cross-sectional dimensions as the main entry with 15 m depth. The main entry had of 19.35 m^3^/s (41000 cfm) of fresh air flowing from the left to the right. The blower fan at the inlet of the push tubing was set to provide 8.02 m^3^/s (17000 cfm) of fresh air into the face area. The exhaust fan at the outlet of the pull tubing drew the diesel exhaust mixture at a rate of 9.44 m^3^/s (20000 cfm) from the face area and released it into the main entry.

For case (1), the push tubing extends into the dead-end entry for approximately 77 m while the pull tubing extended for approximate 67 m. For case (2), the push tubing extended for 70 m into the dead-end and the pull tube extended for approximate 81 m. In case (3), the length of the push tubing remained the same as in case (1) but the pull tubing, in addition to extending for 78 m into the dead-end, curved for an additional 12 m into the face area. In case (4), the length of the push tubing remained the same as in case (2) but the pull tubing, in addition to extending for 78 m into the dead-end, curved for an additional 12 m into the face area. The diameter of both the push and pull tubing was 0.8 m for all the cases and were placed 4.3 m above the ground.

### Assumptions

Based on Zheng’s studies (Zheng and Tien 2008; Zheng et al. 2011), this CFD simulation was conducted under the following assumptions: (1) DPM was treated as gas and the material selected as the representative for DPM was n-octane vapor (C_8_H_18_). The chemical reactions between species are not considered in this study; (2) both air and DPM are incompressible; (3) the flow in the domain is fully turbulent; (4) Both the loader and truck are stationary.

### CFD modelling

Three-dimensional incompressible unsteady turbulent continuity, momentum, and energy equations, along with standard *k*-*ε* turbulent and non-reacting transport Eqs. (2 species, DPM and air) were solved using ANSYS FLUENT CFD software. The species transport model, available in FLUENT, was used to determine the DPM distribution pattern.

Due to the multiple cases covered in this section, all of the boundary conditions are summarized in Table 1. For the LHD, the tailpipe is located at the right rear of the LHD and pointing backward; for the truck, the tailpipe is placed at the right front of the vehicle, pointing to the floor. The parameters for the main ventilation and diesel vehicles are derived from Zheng’s industrial field study (McGinn et al. 2004; Zheng et al. 2010) with only the low emission diesel engines. The detailed meanings of the boundary conditions are presented in other sections and in the FLUENT manual (ANSYS 2014).

**Table 1:** Summary of boundary conditions

	Boundary	Detailed settings
Main ventilation	Inlet	0.65 m/s, normal to boundary; DPM: 0 ppm
	Exit	Pressure outlet (0 Pa)
Diesel equipments	LHD tailpipe	24.1 m/s, normal to boundary; 594 K; DPM, 1.73 ppm
	Truck tailpipe	27.5 m/s, normal to boundary; 644 K; DPM, 2.0 ppm
Walls		No slip, adiabatic walls
Auxiliary ventilation	Push-tube	Inlet: fan (Δ*p* = 481 Pa); outlet: interior
	Pull-tube	Inlet: interior; outlet: fan (Δ*p* = 800 Pa)

In order to achieve accuracy in the simulation results, finer meshes were generated for the area close to diesel engines where high gradients existed. For all the models, about 1.5 million computational elements (cells) were generated. The unsteady flow calculations were made by using time step (Δ*t* = 0.1 s) for the time period of 200 s (3 min and 20 s) for the loading operation.

## Results and discussion

### Long push and short pull tubing system (Case 1)

**Fig. 5:**
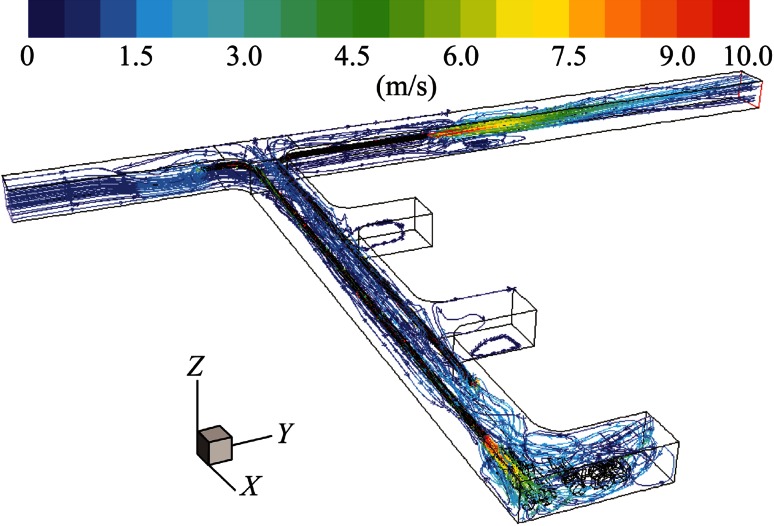
Pathlines colored by velocity magnitude demonstrating general flow features for long push short pull

It can be seen from Fig. 5 that, when the pull tubing was shorter than the push tubing, the fresh air from the push tubing impinged on the rear wall of the dead-end and made a 90° turn to enter the face area. However, only a small portion of that fresh air reached the interior of the face area to ventilate the LHD emission. The remaining portion created a recirculation region in the rear section of the truck and in the frontal portion of the LHD, and then gradually migrated toward the main entry and inlet of the pull tubing. The distant location of the pull tubing made it difficult to remove all the exhaust mixture from the face area in a timely manner.

**Fig. 6:**
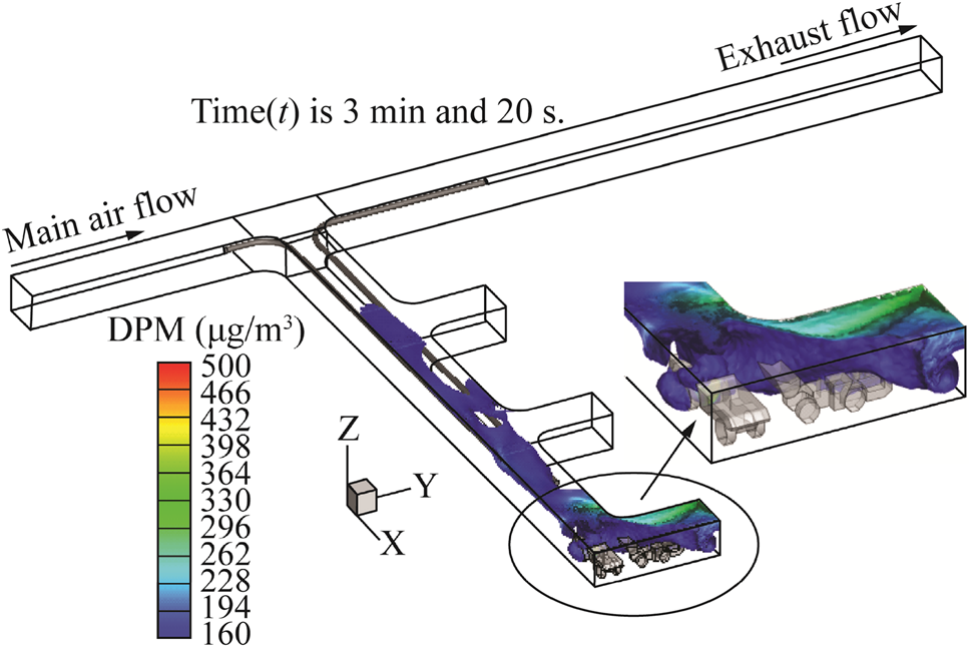
DPM distribution inside the single dead-end entry for long push short pull

The DPM distribution with long push tubing and short pull tubing is shown in Fig. 6. The push tubing was longer than the pull tubing by 10 m. The colored contours represent the diesel exhaust, with the DPM level above the regulation limit of 160 µg/m^3^. The blower fan at the inlet of the push tubing provided 8.02 m^3^/s (17000 cfm) of fresh air to the face area. This low-temperature fresh air mixed and cooled the high-temperature diesel exhausts of the LHD and truck engines and formed a DPM-air mixture. The exhaust fan at the outlet of the pull tubing sucked this exhaust mixture at a rate of 9.44 m^3^/s (20000 cfm) and released it into the main entry. The high concentration DPM completely engulfed the active face area in the dead-end, except for small regions behind the truck and in front of the LHD. Most of the remaining areas of the dead-end were also filled with diesel fumes near the roof region by the end of the loading operation due to the buoyancy effect. The operators of the LHD and the truck should use enclosed cabs to protect themselves from the harmful effects of DPM. This design of long push and short pull tubing system failed to ventilate the active face area effectively.

### Short push and long pull tubing system (Case 2)

**Fig. 7:**
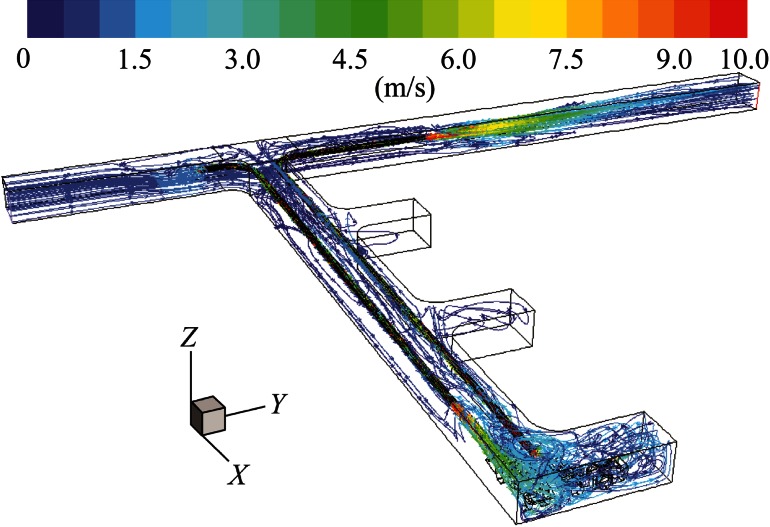
Pathlines colored by velocity magnitude demonstrating general flow features for short push long pull

In this case, the pull tubing was made longer than the push tubing, as shown in Fig. 7. With this modified design, the fresh air flow impinging on the rear wall of the dead-end created a strong vortex-like flow in the presence of pull tubing, as shown in the figure. Again, not enough fresh air reached the interior of the face area. But the closer location of the pull tubing to the face region helped to suck in more tailpipe emission of the LHD.

**Fig. 8:**
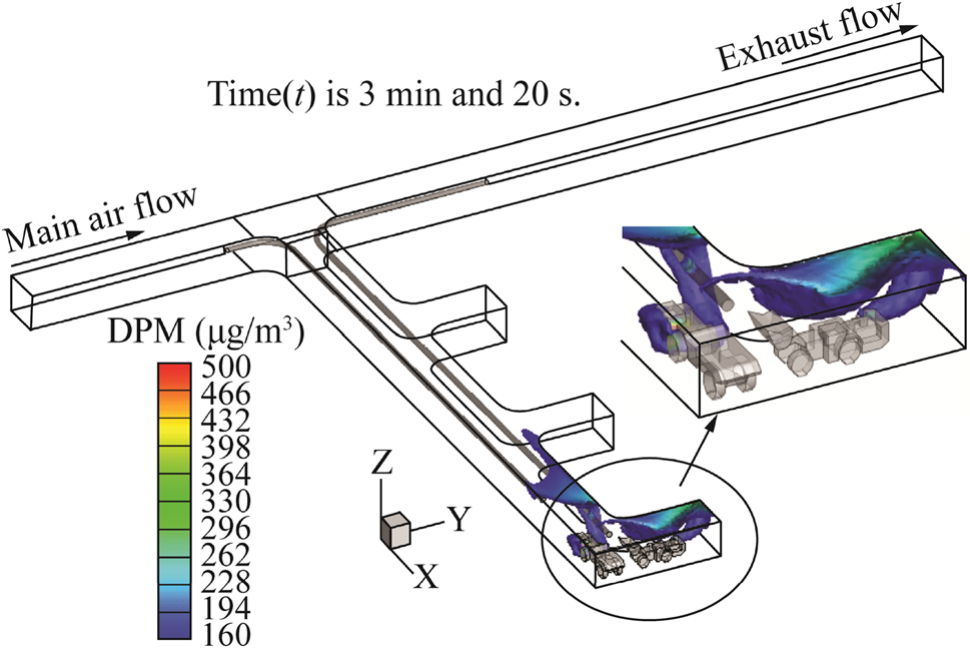
DPM distributions inside the single dead-end entry for short push long pull

Figure 8 shows the DPM distribution in the same dead-end with a short push and long pull tubing system for the LHD-truck loading operation. This modified design of short push and long pull tubing system effectively ventilated the face area, when compared with the long push and short pull tubing ventilation system. The DPM occupied a small region behind the tailpipe of the LHD, a small region around the tailpipe of the truck and the roof region near the face area due to the buoyancy force, as shown by the colored region. The miners working in this colored region should use personal protection instruments. The remaining areas of the dead-end were free of any DPM above the regulatory limit.

### Long push and curved pull tubing system (Case 3)

**Fig. 9:**
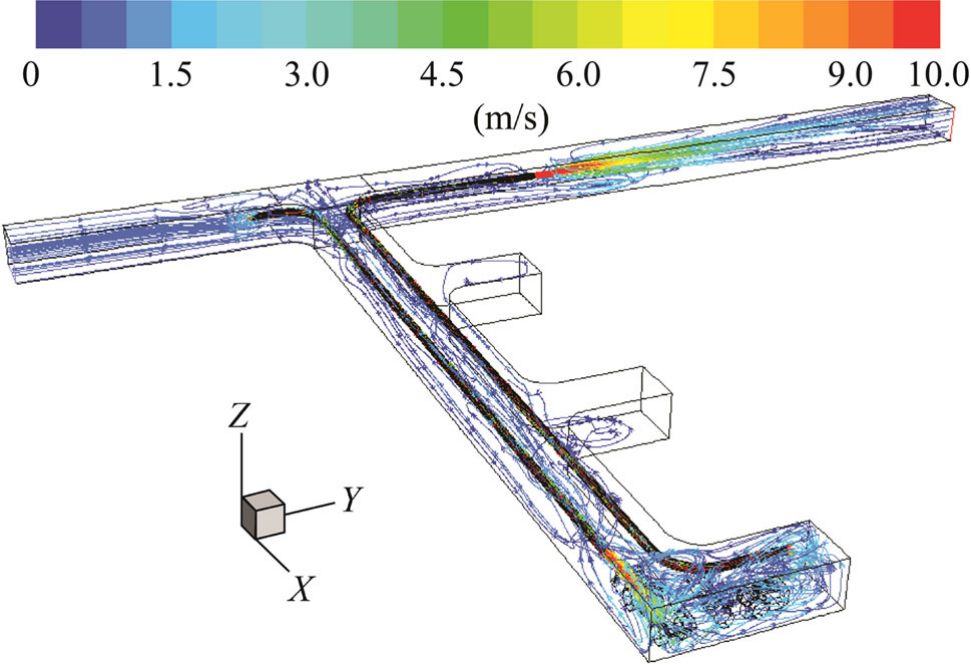
Pathlines colored by velocity magnitude demonstrating general flow features for long push curved pull

It can be seen in Fig. 9 that two recirculation regions were created in the face area: one is around the truck; the other is encircling the LHD. When compared with Fig. 5, there was a large improvement in the ventilation of the face area at the back of LHD around the inlet region of the pull tubing.

**Fig. 10:**
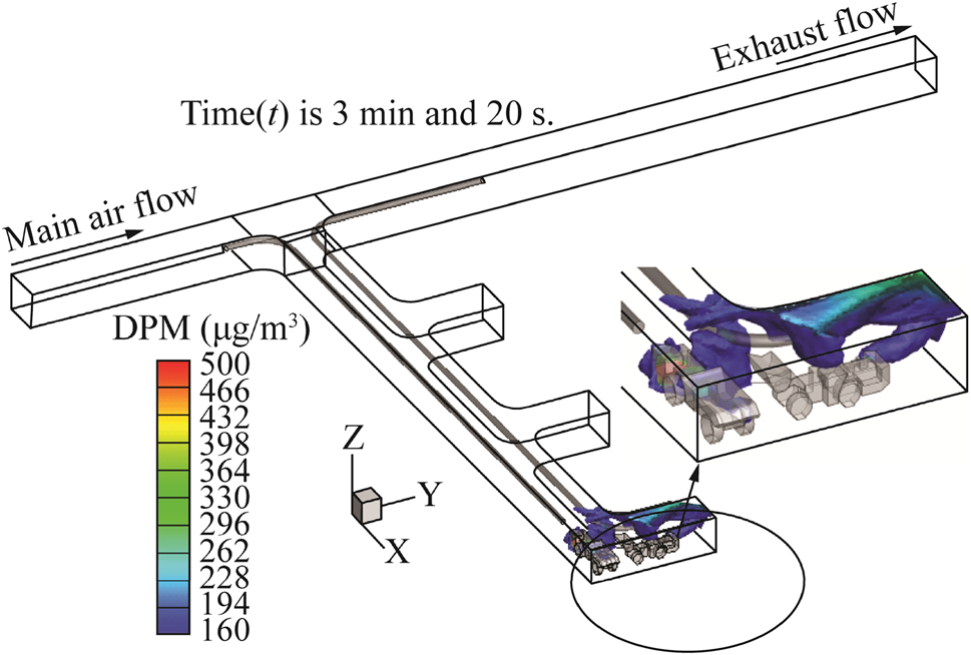
DPM distributions inside the single dead-end entry for long push curved pull

This system is similar to case 1 except that the pull tubing was extended and made to curve inside the face area. The resultant DPM distribution in the dead-end is shown in Fig. 10. When compared with case 1, there was a dramatic improvement in the DPM distribution in the dead-end. There was no high concentration DPM accumulation in the dead-end other than in the face area. However, this system did not perform better than the system in case 2 since there was significant DPM accumulation inside the face area where the LHD was located. It seems that a part of the DPM plume from LHD was missing the inlet of the pull tubing due to the high velocity of air from the push tubing. Miners in the colored region should use personal protection instruments during their working.

### Short push and curved pull tubing system (Case 4)

**Fig. 11:**
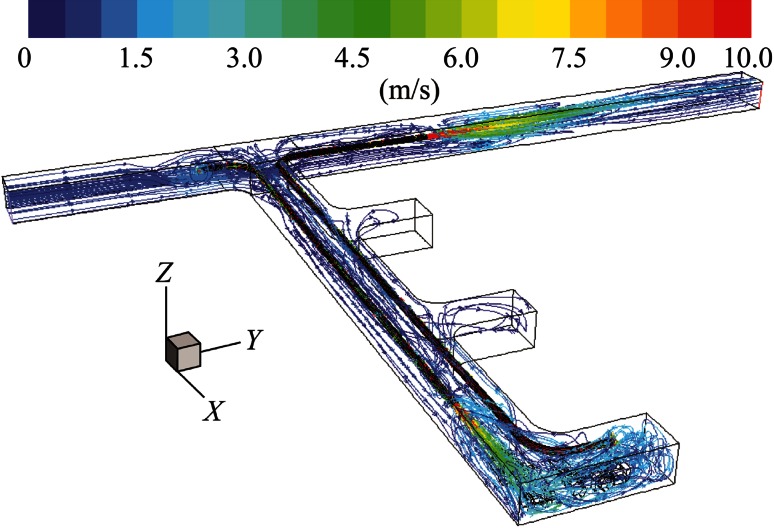
Pathlines colored by velocity magnitude demonstrating general flow features for short push curved pull

As shown in Fig. 11, there was a single large recirculation region which effectively ventilated the entire face area.

**Fig. 12:**
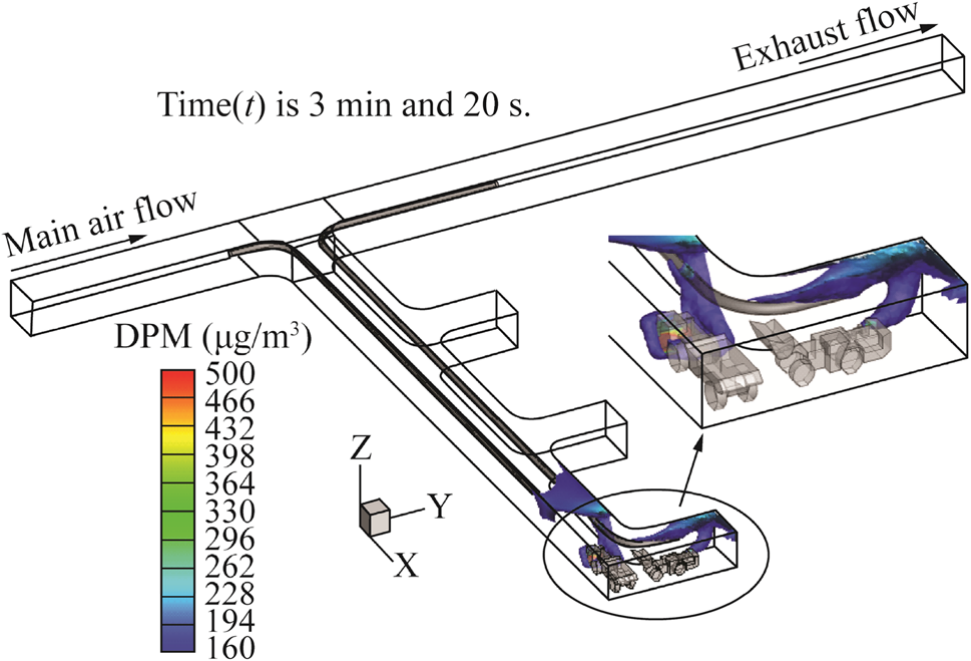
DPM distributions inside the single dead-end entry

The DPM distribution, when a short push and long curved pull tubing auxiliary ventilation system was used for the LHD-truck loading operation, is shown in Fig. 12. This design is similar to case 2 with the short push and long pull tubing design except that the long pull tubing was made to curve for an additional 12 m into the working face area of the dead-end. A comparison of the design of the short push and long curve pull tubing system with short push and long straight pull tubing system showed only a negligible difference for the DPM affected areas near the tailpipe of the LHD and the truck. However, it significantly reduced the DPM accumulation in the roof region of the face area. As mentioned above, miners in the colored regions should use personal protection instruments during their working.

## Comparison of different push–pull tubing designs

**Fig. 13:**
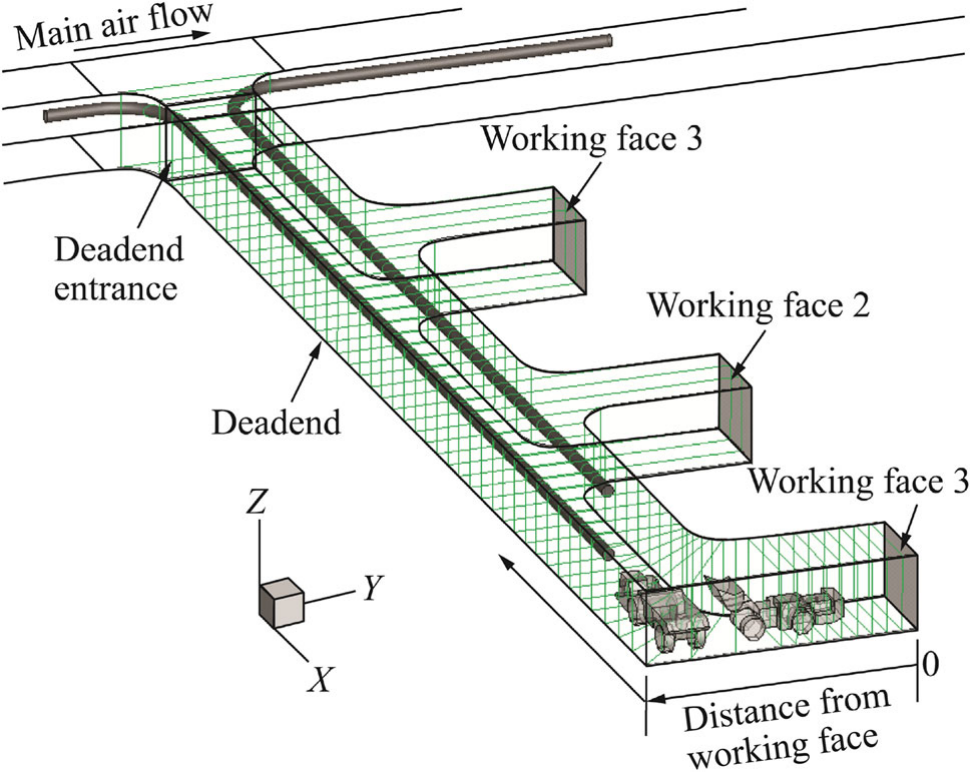
Schematic of the cross-sectional planes inside the dead-end

A comparison of the different designs of the push–pull ventilation systems was made by plotting the area weighted DPM values at cut cross-sectional planes inside the dead-end. The cross-sectional planes are shown in Fig. 13 for the long push and short pull tubing ventilation system. Similar cross-sectional planes were created for other ventilation systems and are not shown here due to space limitations. The performance of each push–pull design system was evaluated based on its DPM dilution capability in the face area.

**Fig. 14:**
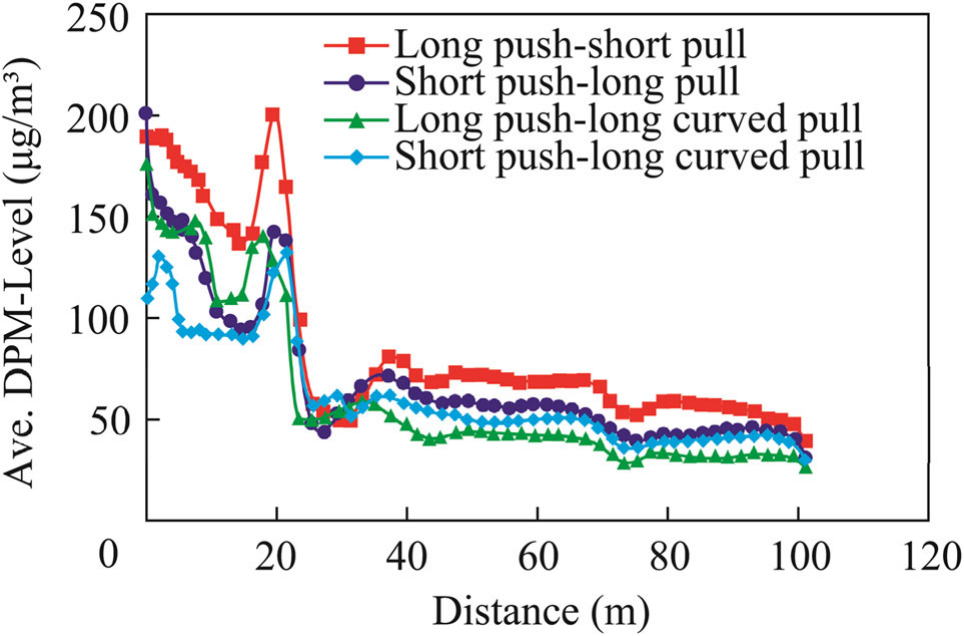
Comparison of average DPM values away from the face

**Fig. 15:**
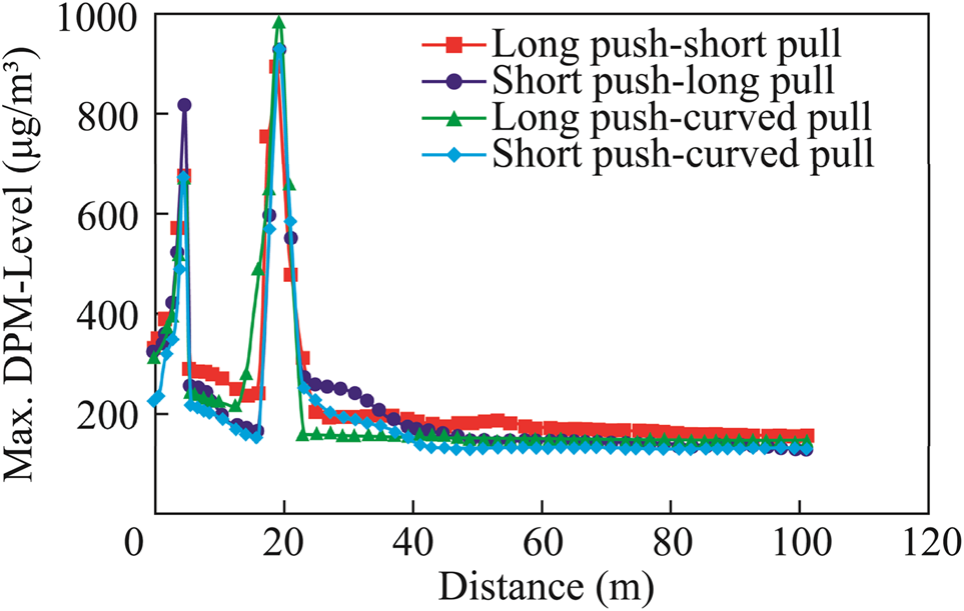
Comparison of the maximum DPM values away from the face

**Fig. 16:**
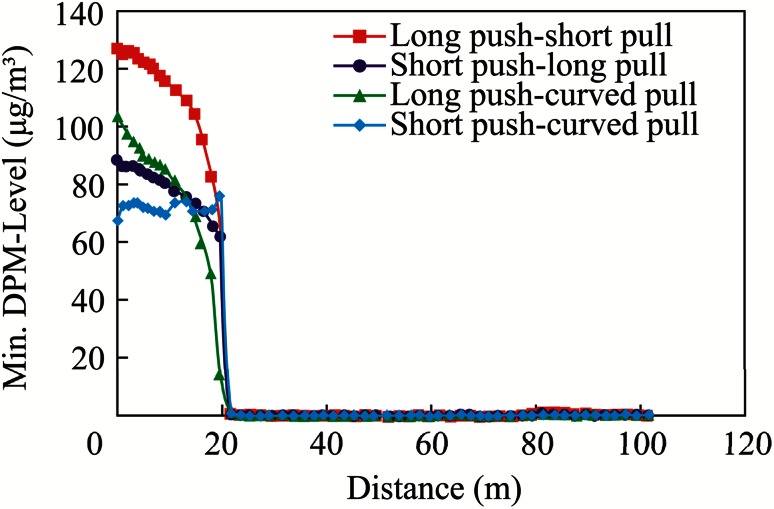
Comparison of the minimum DPM values away from the face

The overall performance evaluation of different push–pull designs was made by plotting the area-weighted average, the maximum, and minimum values of DPM at these cross-sectional planes against the distance from the face, as shown in Figs. 14, 15, and 16. The distance of the cross-sectional planes from the interior face area was evaluated, as shown in Fig. 13. It can be seen from Fig. 14, that case 4, the short push and curved pull tubing system, performed the best with the minimum average DPM value inside the face area, while case 1, the long push and short pull tubing system, performed the worst with the maximum average values inside the face area, when compared with other push–pull deisgns. Although case 3, the long push and curved pull system, resulted in minimum average values in the remaining areas of the dead-end, when compared with other systems, in the important face region where miners were working, case 4, the short push and curved pull tubing design, performed the best.

This fact was further substantiated with the plots of the maximum and minimum value of DPM at the cross-sectional planes, as shown in Figs. 15 and 16. Although the plot of the maximum value (Fig. 15) showed negligible differences in the distribution, the plot of the minimum value (Fig. 16) showed that minimum DPM values were obtained in the face area in case 4, and the short push and curved pull tubing system, was used.

## Conclusions

It was found out by this study that, although clean engines were used in the face area, the ventilation condition can play an essential role in controlling DPM under the regulation requirements. Without proper auxiliary ventilation, the face area and dead-end entry will eventually filled with high DPM fumes.

From the comparison study of these four push–pull ventilation systems, it was concluded that the long push tubing provided powerful airflow in the face region and the DPM air mixture occupied more face region due to the high mixing force. Short push tubing produced better forward airflow momentum to confine the DPM plume in the face region and not over mixing the DPM with airflow. On the contrary, long or curved pull tubing served better DPM removal capacity as it approached high DPM face region and sucked the contaminant air directly out of the working area. Short pull tubing cannot remove DPM effectively as its influence region was too far away from the high DPM regions.

Based on this study, it was concluded that the short push and curved pull tubing system was the best design of all four designs, and effectively ventilated the face area during the truck loading operation. In case of unpractical, the straight short push and long pull tubing system can also have close ventilation result.
